# Cardiovascular safety of exenatide BID: an integrated analysis from controlled clinical trials in participants with type 2 diabetes

**DOI:** 10.1186/1475-2840-10-22

**Published:** 2011-03-16

**Authors:** Robert Ratner, Jenny Han, Dawn Nicewarner, Irina Yushmanova, Byron J Hoogwerf, Larry Shen

**Affiliations:** 1 MedStar Health Research Institute, Hyattsville, MD, USA; 2 Amylin Pharmaceuticals, Inc., San Diego, CA, USA; 3 Eli Lilly and Company and LillyUSA, Indianapolis, IN, USA

**Keywords:** GLP-1 receptor agonist, diabetes, cardiovascular safety

## Abstract

It is important for patients that treatments for diabetes not increase cardiovascular (CV) risk. The objective of this analysis was to examine retrospectively the CV safety of exenatide BID, a GLP-1 receptor agonist approved for treating hyperglycemia in patients with type 2 diabetes not adequately controlled with diet and exercise. Individual participant data was pooled to assess the relative risk (RR) of CV events with exenatide BID versus a pooled comparator (PC) group treated with either placebo or insulin from 12 controlled, randomized, clinical trials ranging from 12-52 weeks. Mean baseline values for HbA_1c _(8.33-8.38%), BMI (31.3-31.5 kg/m^2^), and duration of diabetes (8 y) were similar between groups. Trials included patients with histories of microvascular and/or macrovascular disease. Customized primary major adverse CV events (MACE) included stroke, myocardial infarction, cardiac mortality, acute coronary syndrome, and revascularization procedures. The Primary MACE RR (0.7; 95% CI 0.38, 1.31), calculated by the Mantel-Haenszel method (stratified by study), suggested that exenatide use (vs. PC) did not increase CV risk; this result was consistent across multiple analytic methods. Because the trials were not designed to assess CV outcomes, events were identified retrospectively from a list of preferred terms by physicians blinded to treatment. Other limitations included the low number of CV events, the short duration of trials (≤1 y), and a single active comparator (insulin). The results of these analyses are consistent with those of a recent retrospective analysis of a large insurance database that found that patients treated with exenatide twice daily were less likely to have a CV event than were patients treated with other glucose-lowering therapies.

**Keywords**: GLP-1 receptor agonist, diabetes, cardiovascular safety

## Background

Despite the numerous advancements in glucose-lowering medications in recent years, the incidence of cardiovascular (CV) morbidity and mortality in patients with type 2 diabetes has not consistently decreased [1]. In 2007, a controversial, widely publicized meta-analysis of 42 trials suggested that rosiglitazone, a thiazolidinedione (TZD), was associated with increased risk of myocardial infarction (MI) and death due to CV events [2-6]. At that time, a joint advisory committee meeting concluded that rosiglitazone increased myocardial ischemia and the FDA added a black box warning to the rosiglitazone label [2,7]. Although a non-inferiority CV outcomes trial, RECORD concluded that rosiglitazone did not increase CV morbidity or mortality compared with other glucose-lowering medications [8]; the open-label and unblinded design of RECORD prompted some to question the quality of the data [7]. Following updated meta-analyses and recent congressional inquiry, concerns arose again about the CV safety of rosiglitazone [9-12]. Recently, an advisory committee meeting (July 2010) concluded that rosiglitazone significantly increased CV risk [7].

The risk-to-benefit profile of medications may begin to take on greater importance for future approvals of new drugs [13,14]. Exenatide twice daily (exenatide BID) was the first GLP-1 receptor agonist approved for treatment of hyperglycemia in patients with type 2 diabetes not adequately controlled with diet and exercise, sulfonylurea (SFU), TZD, or metformin (MET; alone, or with an SFU, or TZD). In addition to glycemic control and a low risk of hypoglycemia, exenatide may favorably affect several CV risk factors, such as blood pressure, lipid profiles, and body weight [15-17]. Because the incidence of CV events was low in each of the clinical trials, we undertook this pooled analysis to provide an integrated assessment of the CV safety data from 12 studies with exenatide BID.

## Methods

### Study Selection

For each clinical trial, a protocol was approved by an institutional review board, in accordance with the principles described in the Declaration of Helsinki (World Medical Association 1997). Each of the trials included in this analysis was conducted by Amylin Pharmaceuticals, Inc. and Eli Lilly and Company. Complete efficacy and safety data for these trials have been reported previously [18-29]. Randomized, controlled trials (8 blinded, 4 open-label) that were completed by September 30, 2008 and of at least 12 weeks in duration, that were included in this analysis were designed to compare the efficacy and tolerability of exenatide BID to placebo or insulin (Table 1). They were either placebo-controlled or active-controlled studies where insulin served as the comparator. The studies included in this analysis were not designed to assess CV events and events were not prospectively adjudicated.

**Table 1:** Summary of controlled studies included in CV analysis

				Exenatide†		Placebo or Comparator	
				
Study/Registry Number		Diabetes Management	Duration* (Weeks)	ITT (N)	Exposure (SY)	ITT (N)	Exposure (SY)
**DeFronzo et al, 2005^18^**	**NCT00039013**	Met	30	223	113.8	113	57.8
**Buse et al, 2004^20^**	**NCT00039026**	SFU	30	254	123.2	123	55.1
**Kendall et al, 2005^19^**	**NCT00035984**	Met + SFU	30	486	254.9	247	122.2
**Zinman et al, 2007^24^**	**NCT00099320**	TZD ± Met	16	121	31.7	112	32.3
**Kadowaki et al, 2008^27^**	**NCT00382239**	SFU ± Met	12	111	23.9	40	9.2
**Gao, et al, 2009^26^**	**NCT00324363**	Met ± SFU	16	234	65.5	233	67.3
**Moretto et al, 2008^28^**	**NCT00381342**	D + E	24	155	65.2	77	33.1
**Gill et al, 2010^29^**	**NCT00516074**	Met and/or TZD	12	28	5.8	26	5.7
**Heine et al, 2005^22^**	**NCT00082381**	Met + SFU	26	282	122.5	267	124.6
**Nauck et al, 2007^23^**	**NCT00082407**	Met + SFU	52	253	220.1	248	228.6
**Davis et al, 2007^25^**	**NCT00099333**	SFU or Meg and/or Met	16	33	7.7	16	5.2
**Barnett et al, 2007^21^**	**NCT00099619**	Met or SFU	16‡	136	37.3	127	38.9

**Totals**		--	--	**2,316**	**1,071.6**	**1,629**	**779.9**

D + E = diet and exercise therapy; ITT = Intent-to-Treat Population; Meg = meglitinide; Met = metformin; OAD = oral antidiabetic medications; SFU = sulfonylurea; TZD = thiazolidinedione

*Duration of treatment with randomized study medication

^†^Includes treatment with exenatide 2.5 mcg or 5 mcg BID for duration of study, or 4 weeks of exenatide 5 mcg BID followed by exenatide 10 mcg BID for remainder of study

^‡^NCT00099619 had a crossover design, with 16 weeks per period (exenatide or insulin glargine)

### Analysis population

The current analyses included patients from the intent-to-treat populations (i.e., patients who received at least 1 dose of randomized study medication) of each study (Table 1). Patients in all studies had type 2 diabetes and were treated continuously with exenatide and MET, SFU, or TZD alone or in combination. All patients were 18 to 75 years-of-age, had a HbA_1c _≤11.0%, a body mass index (BMI) of 25 to 45 kg/m^2^, and a history of stable body weight (≤10% change) for at least 3 months. Patients were excluded if they had used weight loss drugs or had evidence of a significant medical condition. Investigators were asked to exclude patients with evidence of active cardiac disease within 1 year prior to the study, i.e., MI, clinically significant arrhythmia, unstable angina, moderate to severe congestive heart failure, coronary artery bypass surgery, or angioplasty. Patients with a >1 year history of MI, transient ischemic attack or large vessel disease or with a history of microvascular disease were eligible for enrollment. All patients provided written informed consent before participation.

### Outcomes

Events were identified by preferred terms according to the Medical Dictionary for Regulatory Activities (MedDRA 11.0). A team of physicians, blinded to treatment, independently reviewed the list of preferred terms prior to the analyses to focus on the terms most likely to represent true events of interest, regardless of occurrence. Blinded adjudication of the CV events was not pre-specified in the study protocols; therefore, events were identified retrospectively using the pre-specified list of preferred terms independent of whether or not the events had occurred. At the time of the occurrence during the original trial, the events were closely reviewed in accordance with normal clinical trial monitoring and follow-up of adverse events, laboratory evaluations, physical examinations, vital signs measurements, with particular attention to serious adverse events.

All data for patients who died were examined to ascertain if the underlying cause was CV in nature based on the preferred term provided and the cases were reviewed in detail. "Sudden deaths" were adjudicated as CV events, in conformance with most large CV outcomes trials [30-35]. The final list of terms was concordant with the FDA list presented at the advisory committee meeting in April 2009 for other antidiabetic agents [36].

### Primary Outcome

The primary outcome was Primary Major Adverse CV Events (MACE); per FDA guidance, it included terms reflective of CV mortality, stroke, myocardial infarction, acute coronary syndrome, and revascularization procedures.

### Secondary Outcome

The secondary CV endpoint included all relevant CV adverse events. This expanded endpoint comprised all terms of the Primary MACE endpoint plus terms for arrhythmia, heart failure (with or without hospitalization), and mechanical-related events. Mechanical-related events were aortic valve disease, aortic valve stenosis, cardiac failure congestive, cardiomegaly, CV disorder, heart valve incompetence, left atrial dilatation, mitral valve incompetence, and tricuspid valve incompetence. Heart failure and mechanical-related adverse events were included in an effort to encompass as many potentially important CV-related events as possible.

### Analysis

A meta-analysis was performed on 12 completed longer-term (3- to 12-month), randomized, placebo- or insulin comparator-controlled trials of exenatide BID, in accordance with the FDA guidelines [37]. Data from approximately 4,000 patients with type 2 diabetes and an average exposure of 24 weeks were included [18-29]. Pooled data from placebo- and insulin-treated patients were compared with pooled data from exenatide-treated patients. The exenatide cohort included participants randomized to receive exenatide BID 2.5 mcg (n = 37), 5 mcg (n = 594), or 10 mcg (n = 1,685). Because of the low number of CV events, the 3 exenatide dose groups were combined.

For the primary analysis, the Relative Risk (RR) of an incident CV event and the corresponding 95% confidence interval (CI) were calculated using Mantel-Haenszel method stratified by study. In order to demonstrate robustness, the RR and its 95% CI were calculated using the following additional methods: pooled RR (without stratification by study and with common continuity correction [i.e., adding 0.5 to all cells if one of the treatment groups had no events]) and Shuster's RR [38] (weights all trials equally and included studies with no events). The Hazard Ratio (HR) was calculated using 2 methods: 1) the Cox proportional hazard model (time to first event) with adjustment for study, and 2) the Andersen-Gill model (recurrent events) with adjustment for study. Ninety-five percent CIs for the RR and HR were provided at a 2-sided significance level of 0.05. Weighted Kaplan-Meier survival curves were generated to show the time to first event and the proportion of patients who were risk-free over time [39]. The time to event was calculated from the first randomized dose to the time of the first cardiac event. Exposure Adjusted Incidence Rate (EAIR) and its 95% CI were calculated using the Exact method [40]. The RR and its 95% CI based on EAIR and event rate were provided [41].

Additional subgroup analyses by age (<65 vs. ≥65), BMI (<30 vs. ≥30), and renal function (normal, mild impairment, and moderate impairment) were provided to assess the effects of these baseline characteristics. SAS 9.2^® ^(Statistical Analysis Software, Cary, NC, USA) was used for all analyses.

## Results

The data included in these analyses represented 1,072 patient-years (PY) exposure with exenatide BID (N = 2,316) and 780 PY exposure with comparators (placebo, n = 971; insulin, n = 658). Demographic and baseline characteristics were similar between treatment groups (Table 2). Participants had similar mean baseline values for HbA_1c _(8.33%-8.38%), BMI (31.3-31.5 kg/m^2^), and duration of diabetes (8 years). Of note, the mean systolic blood pressures at baseline for both cohorts were 131 ± 4 mm Hg and 132 ± 5 mm Hg for the exenatide and the pooled comparator cohorts, respectively. Some patients had a past history of CV disease (including multiple events) and/or microvascular disease (including multiple conditions). From baseline to endpoint, heart rate changes were +0.5 ± 9.8 beats per minute (bpm) for exenatide and +0.1 ± 9.2 bpm for the pooled comparators (mean ± SD).

**Table 2:** Baseline characteristics and demographics

Baseline Characteristics	Exenatide (N = 2,316)	Pooled Comparator (N = 1,629)
**Gender, M/F (%)**	56/44	53/47
**Race (%)**		
Caucasian	64	65
Black	6	4
Hispanic	11	10
Asian	19	20
Other	1	1
**Age (years)**	56 ± 10	56 ± 10
≥65 years (%)	21	20
**Duration of Diabetes (years)**	8 ± 6	8 ± 6
≥10 years (%)	32	35
**Weight (kg)**	89.1 ± 20.3	87.8 ± 19.8
**Body Mass Index (BMI [kg/m^2^])**	31.5 ± 5.6	31.3 ± 5.4
BMI <30 kg/m^2 ^(%)	44	45
BMI ≥30 kg/m^2 ^(%)	56	55
**HbA_1c _(%)**	8.33 ± 1.06	8.38 ± 1.07
**Systolic Blood Pressure (SBP [mm Hg])**	131 ± 4	132 ± 5
**Diastolic Blood Pressure (DBP [mm Hg])**	79 ± 2	79 ± 1
**Heart Rate (beats per minute)**	75 ± 9	75 ± 9
Renal Function Impairment* (%)		
None	86	86
Mild	13	14
Moderate	1	1

Data are mean ± SD unless otherwise noted. Percentages may not add to 100 due to rounding

*Renal function is defined based on creatinine clearance (CrCl) as calculated by the Cockcroft-Gault equation: normal, CrCl >80 mL/min; mild impairment, CrCl >50-80 mL/min; moderate impairment, CrCl >30-50 mL/min

Incidence rates of Primary MACE were similar for 5 mcg (0.9%) and 10 mcg (1.1%) exenatide dose groups, and no events were observed in the 2.5 mcg group. Therefore, data from all exenatide doses (2.5 mcg, 5 mcg, 10 mcg) were pooled in the analyses. The EAIR for exenatide treatment group was 18.73 per 1,000 patient-years versus 23.17 per 1,000 patient-years for the pooled comparator group (Table 3). Overall, 26 participants experienced serious adverse CV events: 15 (0.6%) in the exenatide group and 11 (0.7%) in the pooled comparator group (data not shown). Four fatal CV events occurred: 2 in the exenatide group (MI, atrial fibrillation) and 2 in the comparator group (MI, cerebrovascular accident).

**Table 3:** Incidence rates and exposure-adjusted incidence rates for primary MACE and secondary CV endpoints

	Primary MACE Endpoint		Secondary MACE Endpoint	
	
	Exenatide (N = 2,316)	Pooled Comparator (N = 1,629)	Exenatide (N = 2,316)	Pooled Comparator (N = 1,629)
**Primary Analyses**				

**Incidence (n)**	20	18	46	42
**Incidence (n/N)**	0.009	0.011	0.020	0.026
RR (95% CI)	0.70 (0.38, 1.31)		0.69 (0.46, 1.03)	

**Secondary Analysis**				

**EAIR (per 1,000 years)**	18.73	23.17	43.37	54.37
RR (95% CI)	0.81 (0.43, 1.53)		0.80 (0.53, 1.21)	
**Event Rate (per 1,000 years)**	22.40	28.21	54.13	66.67
RR (95% CI)	0.79 (0.45, 1.42)		0.81 (0.56, 1.18)	

CI = confidence interval; CV = cardiovascular; EAIR = exposure-adjusted incidence rate; MACE = major adverse cardiovascular events; RR = risk ratio

RR for the incidence was calculated using the Mantel-Haenszel estimate with study as a stratification factor

EAIR and event rate were calculated based on the Exact method, with corresponding RR calculated using the log-normal approximation

**Figure 1:**
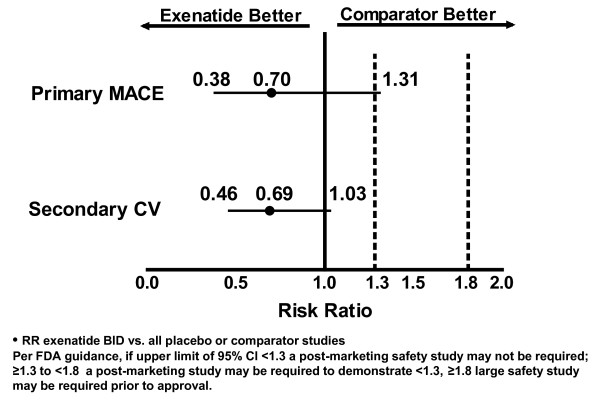
**Risk of primary MACE and secondary CV endpoints with exenatide BID relative to pooled comparators**.

**Figure 2:**
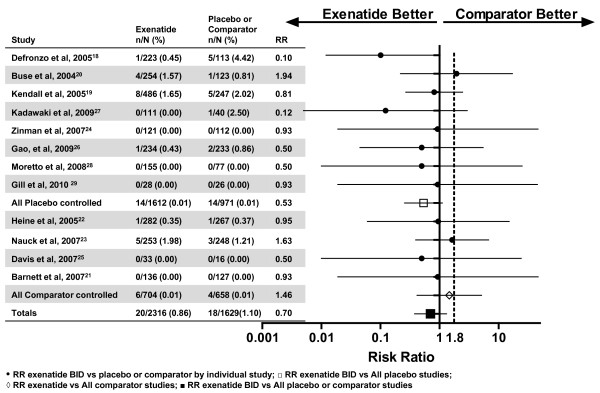
**Incidence and RR for primary MACE with exenatide use by individual study**.

**Figure 3:**
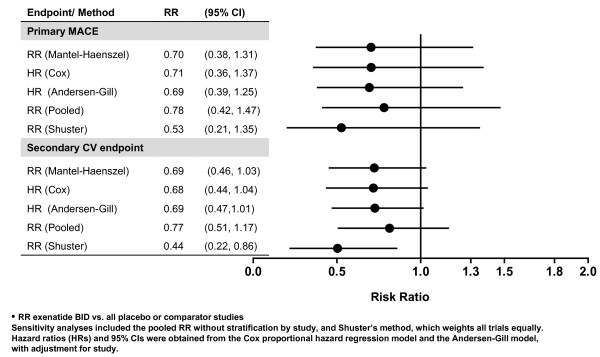
**Forest plot of CV end points by statistical method**.

**Figure 4:**
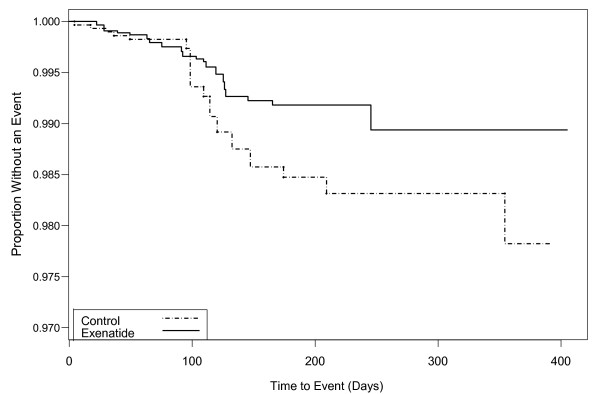
**Weighted Kaplan Meier plot for subjects without a primary MACE event by treatment in controlled studies of exenatide BID**.

The point estimates for Primary MACE and Secondary CV endpoints between exenatide BID and the pooled comparator were both 0.7 (the upper limits of the 95% CIs were 1.3 and 1.0, respectively), in favor of exenatide (Figure 1). These results suggest that exenatide did not increase the CV risk. For the individual studies, the point estimates for RR were <1.0 (favoring exenatide) for 10 of 12 long-term controlled studies (Figure 2). In addition, the RRs and 95% CIs for the Primary MACE were consistent across multiple methods of analysis (including sensitivity analyses of RR based on incidence and RR based on EAIR event rate), with point estimates ranging from 0.5 to 0.8, and the upper limit of the 95% CIs ranging from 1.3 to 1.5 (Figure 3). Similar point estimates were observed for the broader secondary CV endpoint; however, the upper limit of the 95% CI was <1.3 for all methods (Figure 3). A weighted Kaplan-Meier plot shows that a significantly higher percentage of exenatide-treated patients than pooled comparator-treated patients were free of a primary MACE event over 1 year (*P *< 0.0001; Figure 4).

## Discussion

### Assessing the CV Risk of Glucose-Lowering Therapies

One of the strengths of this meta-analysis is the use of individual participant data from each of the trials, in contrast with a typical meta-analysis in which summary statistics for individual studies are used. Another strength was the use multiple methods of analysis, some of which allowed for the inclusion of studies with zero CV event rates. The results of this meta-analysis provide a preliminary assessment of the CV risk associated with exenatide treatment across trials used for registration. Because these findings are not conclusive, a prospective, adequately-powered, adjudicated, CV outcome study (NCT01144338) of the investigational once-weekly formulation of exenatide was initiated.

Applying the FDA guidance on how to assess CV safety in the development of treatments for type 2 diabetes to this analysis, the HR point estimates were both <1 for the Primary MACE and Secondary CV endpoints resulting from the lower observed incidence of CV events with exenatide BID compared to the control group [37]. These results statistically excluded a 1.8-fold increase of the CV risk by exenatide and were consistent with the FDA guideline that state that the upper limit of a 2-sided 95% CI for new diabetes therapies should be <1.8. In fact, the results suggest that exenatide treatment may improve CV outcomes, although this effect failed to reach statistical significance. Our results are consistent with those of a recent retrospective analysis of a large insurance database in which the 39,275 patients treated with exenatide twice daily were found to be significantly less likely to have a CV event than were the 381,218 patients treated with other glucose-lowering therapies (HR 0.81, 95% CI 0.68-0.91, *P *= 0.01) [42]. Although the results of the current meta-analysis suggest that there may be an association between exenatide and improved CV outcomes, data from multi-year, randomized, controlled, adequately-powered clinical trials with prospective blinded adjudication of CV events are needed to evaluate whether exenatide has cardio-protective effects.

Despite the increased *relative risk *of CV disease in diabetes, the *absolute risk *may only be reduced when multifactorial treatment strategies are used [43-45]; therefore, the CV risk associated with glucose-lowering agents is not easily ascertained. Even in randomized, controlled trials that included CV outcomes as part of the endpoint, the outcomes may be difficult to interpret. The University Group Diabetes Program (UGDP) suggested treatment with tolbutamide, a first generation SFU, was associated with increased risk of negative CV outcomes [46]. Despite the limitations of UGDP, the implication that SFUs were associated with some risk remained until the results of the landmark UKPDS were available [47]. From UKPDS, it was evident that SFU or MET monotherapy was associated with reduced CV risk; however, treatment with the combination of SFU and MET was associated with increased CV risk [48]. Demonstration of the impact of glycemic control on the risk of acute MI was limited by the relatively low absolute MI event rate, but long-term follow-up studies of UKPDS and DCCT found that intense glycemic control reduced major CV events [31,49,50].

Four large trials have analyzed the effects of glucose lowering on CV risk [30,31,35,47,49,51]. The UKPDS studied patients early in the course of disease and found that intensive therapy was associated with a reduction in CV events, especially in the small cohort of obese patients who were treated with metformin [47,49]. The 3 large trials (ADVANCE, ACCORD, and VADT) in patients with longer duration of disease generally had HRs less than 1.0 with intensive therapy, but none achieved statistical significance [30,35,51]. The systematic review of these 4 trials in a pooled analysis did show a reduction in CV disease (RR 0.90, 95% CI 0.83-0.98) and coronary heart disease (RR 0.89, 95% CI 0.81-0.96), but not in stroke, coronary heart failure, CV disease mortality, or all-cause mortality [52]. ACCORD and VADT reported that mortality was associated with hypoglycemia [35,51,53]. In fact, ACCORD was discontinued because of this increased mortality. Hypoglycemia in each of the arms of the ACCORD trial was associated with increased mortality, though hypoglycemia did not explain the increased mortality risk in the intensive arm [53]. Recently, lower HbA_1C _values in the intensive arm were reported to be associated with reduced CV disease events [54] and difficulty in lowering HbA_1C _below 7% in the intensive group may actually have been the best predictor of CV events [54]. None of the trials in patients with longer duration of disease demonstrated a clear adverse (or beneficial) effect of any particular glucose-lowering medication, although the trials were not designed to answer this question. However, a reasonable interpretation is that there may be small to modest benefits on CV disease events with glucose lowering as long as such glucose lowering can be achieved without increased risk for hypoglycemia. In addition, the difficulty in understanding the results of the recent CV trials may be due, in part, to the declining incidence rate of CV events in patients with diabetes. The declining event rates observed in the most recent CV studies may suggest that improvements to CV risk may be due to other temporal phenomena. Resolving the problem of residual risk is increasingly difficult to prove experimentally because the low absolute risk of an event requires large numbers of subjects to be followed for a long period of time with a disease that progresses and requires evolving therapeutic intervention.

### Potential CV Effects of GLP-1 Receptor Agonists

Potential mechanisms of CV disease protection by GLP-1 in humans have been proposed but not established. One possibility is glycemic control, given the positive correlation between HbA_1C _and CV events [55,56]. Another possibility is the association of GLP-1 receptor agonist treatment with weight stability or reduction. Although the effects of weight gain in treated patients with type 2 diabetes have not been thoroughly investigated, an observational study showed that CV disease risk in patients with type 2 diabetes increased with increasing BMI [57]. Although the majority of patients with type 2 diabetes are overweight and at greater risk of CV disease than are patients without type 2 diabetes [58], most conventional diabetes medications are weight neutral or induce weight gain [59,60].

Improving glycemic control, supporting weight loss, and minimizing hypoglycemia are the clinical priorities in the management of most patients with type 2 diabetes [61]. During the last decade, exenatide BID therapy has been consistently associated with improvements in glycemic control coupled with weight loss in clinical and observational studies of patients with type 2 diabetes [62,63]. Importantly, exenatide therapy does not increase the risk of severe hypoglycemia when used in the absence of agents commonly associated with hypoglycemia, primarily SFU and insulin. In several small studies, insulin-induced hypoglycemia was associated with lengthened QT intervals, which may increase the risk of arrhythmia [64,65]. It is possible that the low risk of hypoglycemia with GLP-1 receptor agonist treatments may contribute to a lower risk of CV events.

Exenatide therapy has been associated with reductions in multiple cardiovascular risk factors. A pooled analysis of randomized controlled clinical trials of exenatide BID demonstrated a significant reduction in systolic blood pressure with exenatide therapy compared with placebo or insulin therapy [66]; a similar result was obtained in a recent randomized controlled clinical trial [67]. In open-label extension studies lasting up to 3 years, exenatide BID treatment resulted in improved glycemic control accompanied by moderate weight loss and improvements in BP, cholesterol levels, inflammatory markers, and insulin resistance for some patients [15-17]. Improvements in postprandial lipidemia associated with exenatide treatment were identified in a 1-year open-label study that compared the efficacies of exenatide and insulin glargine [68,69]. Compared with insulin-treatment, significant decreases in post-prandial triglycerides, free fatty acids, HDL-C, VLDL-C, and Apo B48 were observed with exenatide treatment. No between-group differences were found in postprandial total cholesterol, LDL-C, ApoA1, ApoA2, Apo B100, or ApoC3, although exenatide reduced the post-prandial oxidative stress markers P-malondialdehyde and oxidized LDL [68]. Reductions in high-sensitivity C-reactive protein and increases in total adiponectin were also observed [69]. The latter changes were not statistically dependent on changes in fat mass or body weight [69].

Exenatide therapy has been associated with 1 or 2 bpm increases in heart rate in individual clinical trials [29,67]. In this analysis, mean heart rate changes were +0.5 bpm for exenatide and +0.1 bpm for the pooled comparators. The clinical importance of small increases in heart rate is unclear. The majority of epidemiological studies in patients with diabetes have studied the effects of 10 bpm increases in heart rate from any cause, which are associated with increased CV risk [70,71].

No effects of exenatide BID on cardiac repolarization (QT/QTc interval) have been observed in preclinical toxicology studies or during clinical studies of exenatide BID in patients with type 2 diabetes. A thorough QT study in healthy volunteers demonstrated that, compared with placebo treatment, exenatide BID treatment was not associated with clinically significant QTc prolongation [72]. A thorough QT study for an investigational, long-acting formulation of exenatide is ongoing.

Extrapolating from pre-clinical studies, it is possible that GLP-1 receptors located in the heart and vasculature may play a protective role with respect to CV disease [73,74]. In animal models, GLP-1 reduced infarct size after coronary artery ischemia [75], improved left ventricular ejection volume in heart failure [73], improved glucose uptake in the myocardium [74], and induced nitric oxide-independent vasorelaxation in the endothelium [74]. Recent studies evaluated treatment with a GLP-1 receptor agonist in rodent models of severe hypertension or congestive heart failure. Dahl salt-sensitive rats fed a high-salt diet were found to have less hypertension, renal dysfunction, and mortality after 4 weeks of continuous therapy with a GLP-1 analog than were those without treatment [76]. Similarly, rats with heart failure after coronary artery ligation that were treated with a GLP-1 analog demonstrated improved cardiac function, cardiac dimension, exercise capacity, and survival compared to untreated rats [77]. In humans, there is limited evidence that GLP-1 improves left ventricular ejection fraction, reperfusion, or functional status in patients with heart failure or MI [78,79]. Further studies are warranted in patients with specific types of CV disease to understand if the latter effects and those on the endothelium are seen in humans. Studies of the CV effects of different formulations of GLP-1 receptor agonists, including an oral formulation, are also justified to determine whether similar results are seen in all members of this drug class [80].

### Limitations

Major limitations of this analysis were the inclusion of studies of short duration, lack of complete data on CV history, and the lack of pre-specified and blinded adjudication of the CV events. In addition, the incidence of CV events was low in the individual trials. As with all meta-analyses, the current meta-analysis was retrospective in nature. Pooling the placebo group with a single active-comparator group is another potential limitation of this analysis. The rationale for pooling the placebo- and active-comparator patients was to provide greater power for this analysis by increasing the sample size and the number of observed outcomes.

## Conclusions

On the basis of this integrated analysis of 3,945 participants representing 1,070 patient-years of exenatide exposure and 780 patient-years of PC exposure, no increase in CV risk was associated with use of exenatide BID (vs. PC) in patients with type 2 diabetes participating in clinical trials. This analysis, conducted in compliance with FDA guidance on the subject, provides insight into the CV safety of exenatide treatment.

## Competing interests

RR has received research support during the last 5 years from Amylin Pharmaceuticals, Inc., Eli Lilly & Company, NovoNordisk, GSK, Roche, and Sanofi Aventis; he is not an employee or stock-holder of any of these companies and does not have any other financial competing interests. JH, DN, IY, and LS were employees and stockholders of Amylin Pharmaceuticals, Inc. at the time of this analysis. BJH is an employee and stockholder of Eli Lilly & Co. Amylin Pharmaceuticals, Inc. and holds the patent for synthetic Exendin-4 (exenatide).

## Authors' contributions

RR reviewed and interpreted the data, requested additional analysis, and critically reviewed the manuscript; JH participated in the acquisition and statistical analysis of the data and reviewed the manuscript; DN participated in analysis and interpretation of the data and drafted the manuscript; IY participated in design of the analysis, data interpretation, and critical review of the manuscript; BJH participated in analysis and interpretation of the data, requested additional analysis, and drafted sections of the manuscript; LS contributed to the conception and design of the analysis, interpreted the analyzed data, and critically reviewed the manuscript. All authors read and approved the final manuscript.

## References

[B1] Buse JB Ginsbert HN Bakris GL Clark N Costa F Eckel R Fonseca V Gerstein H Grundy S Nesto R Pignone MP Plutzky J Porte D Redbert R Stitzel KF Stone NJ Primary prevention of cardiovascular diseases in people with diabetes mellitus: a scientific statement from the American Heart Association and the American Diabetes Association Diabetes Care 2007 30 162 172 10.2337/dc07-9917 17192355

[B2] Nissen SE Wolski K Effect of rosiglitazone on the risk of myocardial infarction and death from cardiovascular causes N Engl J Med 2007 356 2457 2471 10.1056/NEJMoa072761 17517853

[B3] Cleland JG Atkin SL Thiazolidinediones, deadly sins, surrogates, and elephants Lancet 2007 370 1103 1104 10.1016/S0140-6736(07)61488-3 17905146

[B4] Diamond GA Bax L Kaul S Uncertain effects of rosiglitazone on the risk for myocardial infarction and cardiovascular death Ann Intern Med 2007 147 578 581 17679700 10.7326/0003-4819-147-8-200710160-00182

[B5] Mulrow CD Cornell J Localio AR Rosiglitazone: a thunderstorm from scarce and fragile data Ann Intern Med 2007 147 585 587 17938398 10.7326/0003-4819-147-8-200710160-00013

[B6] Psaty BM Furberg CD Rosiglitazone and cardiovascular risk N Engl J Med 2007 356 2522 2524 10.1056/NEJMe078099 17517854

[B7] Rosen CJ Revisiting the rosiglitazone story--lessons learned N Engl J Med 2010 363 803 806 10.1056/NEJMp1008233 20660395

[B8] Home PD Pocock SJ Beck-Nielsen H Curtis PS Gomis R Hanefeld M Jones NP Komajda M McMurray JJ Rosiglitazone evaluated for cardiovascular outcomes in oral agent combination therapy for type 2 diabetes (RECORD): a multicentre, randomised, open-label trial Lancet 2009 373 2125 2135 10.1016/S0140-6736(09)60953-3 19501900

[B9] Nissen SE Wolski K Rosiglitazone revisited: an updated meta-analysis of risk for myocardial infarction and cardiovascular mortality Arch Intern Med 2010 170 1191 1201 10.1001/archinternmed.2010.207 20656674

[B10] Graham DJ Ouellet-Hellstrom R MaCurdy TE Ali F Sholley C Worrall C Kelman JA Risk of acute myocardial infarction, stroke, heart failure, and death in elderly Medicare patients treated with rosiglitazone or pioglitazone JAMA 2010 304 411 418 10.1001/jama.2010.920 20584880

[B11] Nissen SE Setting the RECORD Straight JAMA 2010 303 1194 1195 10.1001/jama.2010.333 20332408

[B12] Juurlink DN Rosiglitazone and the case for safety over certainty JAMA 2010 304 469 471 10.1001/jama.2010.954 20584879

[B13] Drucker DJ Sherman SI Gorelick FS Bergenstal RM Sherwin RS Buse JB Incretin-based therapies for the treatment of type 2 diabetes: evaluation of the risks and benefits Diabetes Care 2010 33 428 433 10.2337/dc09-1499 20103558 PMC2809297

[B14] Parks M Rosebraugh C Weighing risks and benefits of liraglutide-the FDA's review of a new antidiabetic therapy N Engl J Med 2010 362 774 777 10.1056/NEJMp1001578 20164475

[B15] Blonde L Klein EJ Han J Zhang B Mac SM Poon TH Taylor KL Trautmann ME Kim DD Kendall DM Interim analysis of the effects of exenatide treatment on A1C, weight and cardiovascular risk factors over 82 weeks in 314 overweight patients with type 2 diabetes Diabetes Obes Metab 2006 8 436 447 10.1111/j.1463-1326.2006.00602.x 16776751

[B16] Ratner RE Maggs D Nielsen LL Stonehouse AH Poon T Zhang B Bicsak TA Brodows RG Kim DD Long-term effects of exenatide therapy over 82 weeks on glycaemic control and weight in over-weight metformin-treated patients with type 2 diabetes mellitus Diabetes Obes Metab 2006 8 419 428 10.1111/j.1463-1326.2006.00589.x 16776749

[B17] Klonoff DC Buse JB Nielsen LL Guan X Bowlus CL Holcombe JH Wintle ME Maggs DG Exenatide effects on diabetes, obesity, cardiovascular risk factors and hepatic biomarkers in patients with type 2 diabetes treated for at least 3 years Curr Med Res Opin 2008 24 275 286 18053320 10.1185/030079908x253870

[B18] DeFronzo RA Ratner RE Han J Kim DD Fineman MS Baron AD Effects of exenatide (exendin-4) on glycemic control and weight over 30 weeks in metformin-treated patients with type 2 diabetes Diabetes Care 2005 28 1092 1100 10.2337/diacare.28.5.1092 15855572

[B19] Kendall DM Riddle MC Rosenstock J Zhuang D Kim DD Fineman MS Baron AD Effects of exenatide (exendin-4) on glycemic control over 30 weeks in patients with type 2 diabetes treated with metformin and a sulfonylurea Diabetes Care 2005 28 1083 1091 10.2337/diacare.28.5.1083 15855571

[B20] Buse JB Henry RR Han J Kim DD Fineman MS Baron AD Effects of exenatide (exendin-4) on glycemic control over 30 weeks in sulfonylurea-treated patients with type 2 diabetes Diabetes Care 2004 27 2628 2635 10.2337/diacare.27.11.2628 15504997

[B21] Barnett AH Burger J Johns D Brodows R Kendall DM Roberts A Trautmann ME Tolerability and efficacy of exenatide and titrated insulin glargine in adult patients with type 2 diabetes previously uncontrolled with metformin or a sulfonylurea: A multinational, randomized, open-label, two-period, crossover noninferiority trial Clin Ther 2007 29 2333 2348 10.1016/j.clinthera.2007.11.006 18158075

[B22] Heine RJ Van Gaal LF Johns D Mihm MJ Widel MH Brodows RG Group ftGS Exenatide versus insulin glargine in patients with suboptimally controlled type 2 diabetes: a randomized trial Ann Intern Med 2005 143 559 569 16230722 10.7326/0003-4819-143-8-200510180-00006

[B23] Nauck MA Duran S Kim D Johns D Northrup J Festa A Brodows B Trautmann M A comparison of twice-daily exenatide and biphasic insulin aspart in patients with type 2 diabetes who were suboptimally controlled with sulfonylurea and metformin: a non-inferiority study Diabetologia 2007 50 259 267 10.1007/s00125-006-0510-2 17160407

[B24] Zinman B Hoogwerf BJ Durán Garcia S Milton DR Giaconia JM Kim DD Trautmann ME Brodows RG The effect of adding exenatide to a thiazolidinedione in suboptimally controlled type 2 diabetes Ann Intern Med 2007 146 477 485 17404349 10.7326/0003-4819-146-7-200704030-00003

[B25] Davis SN Johns D Maggs D Xu H Northrup JH Brodows RG Exploring the substitution of exenatide for insulin in patients with type 2 diabetes treated with insulin in combination with oral antidiabetes agents Diabetes Care 2007 30 2767 2772 10.2337/dc06-2532 17595353

[B26] Gao Y Yoon KH Chuang LM Mohan V Ning G Shah S Jang HC Wu TJ Johns D Northrup J Brodows R Efficacy and safety of exenatide in patients of Asian descent with type 2 diabetes inadequately controlled with metformin or metformin and a sulphonylurea Diabetes Res Clin Pract 2009 83 69 76 10.1016/j.diabres.2008.09.037 19019476

[B27] Kadowaki T Namba M Yamamura A Sowa H Wolka AM Brodows RG Exenatide exhibits dose-dependent effects on glycemic control over 12 weeks in Japanese patients with suboptimally controlled type 2 diabetes Endocr J 2009 56 415 424 10.1507/endocrj.K08E-296 19194050

[B28] Moretto TJ Milton DR Ridge TD MacConell LA Okerson T Wolka AM Brodows RG Efficacy and tolerability of exenatide monotherapy over 24 weeks in antidiabetic drug-naive patients with type 2 diabetes: a randomized, double-blind, placebo-controlled, parallel-group study Clin Ther 2008 30 1448 1460 10.1016/j.clinthera.2008.08.006 18803987

[B29] Gill A Hoogwerf BJ Burger J Bruce S Macconell L Yan P Braun D Giaconia J Malone J Effect of exenatide on heart rate and blood pressure in subjects with type 2 diabetes mellitus: a double-blind, placebo-controlled, randomized pilot study Cardiovasc Diabetol 2010 9 6 10.1186/1475-2840-9-6 20109208 PMC2823663

[B30] ADVANCE Collaborative Group Intensive blood glucose control and vascular outcomes in patients with type 2 diabetes New Engl J Med 2008 358 2560 2572 10.1056/NEJMoa0802987 18539916

[B31] UK Prospective Diabetes Study Group Intensive blood-glucose control with sulphonylureas or insulin compared with conventional treatment and risk of complications in patients with type 2 diabetes (UKPDS 33) Lancet 1998 352 837 853 10.1016/S0140-6736(98)07019-6 9742976

[B32] Davis BR Cutler JA Gordon DJ Furberg CD Wright JT Jr Cushman WC Grimm RH LaRosa J Whelton PK Perry HM Alderman MH Ford CE Oparil S Francis C Proschan M Pressel S Black HR Hawkins CM Rationale and design for the Antihypertensive and Lipid Lowering Treatment to Prevent Heart Attack Trial (ALLHAT). ALLHAT Research Group Am J Hypertens 1996 9 342 360 10.1016/0895-7061(96)00037-4 8722437

[B33] Farmer JA Gotto AM Jr The Heart Protection Study: expanding the boundaries for high-risk coronary disease prevention Am J Cardiol 2003 92 3i 9i 10.1016/S0002-9149(03)00503-4 12867249

[B34] Scandinavian Simvastatin Study Group Randomised trial of cholesterol lowering in 4444 patients with coronary heart disease: the Scandinavian Simvastatin Survival Study (4S) Lancet 1994 344 1383 1389 7968073

[B35] ACCORD Study Group Effects of intensive glucose lowering in type 2 diabetes N Engl J Med 2008 358 2545 2559 10.1056/NEJMoa0802743 18539917 PMC4551392

[B36] Food and Drug Administration Official transcript Endocrinologic and Metabolic Drugs Advisory Committee meeting, April 1, 2009 2009 Silver Spring, MD http://www.fda.gov/downloads/AdvisoryCommittees/CommitteesMeetingMaterials/Drugs/EndocrinologicandMetabolicDrugsAdvisoryCommittee/UCM151169.pdf updated 02/17/2010

[B37] Food and Drug Administration Guidance for industry: diabetes mellitus-evaluating cardiovascular risk in new antidiabetic therapies to treat type 2 diabetes 2008 Silver Spring, MD http://www.fda.gov/downloads/Drugs/GuidanceComplianceRegulatoryInformation/Guidances/ucm071627.pdf cited 2009 August 27

[B38] Shuster JJ Jones LS Salmon DA Fixed vs random effects meta-analysis in rare event studies: the rosiglitazone link with myocardial infarction and cardiac death Stat Med 2007 26 4375 4385 10.1002/sim.3060 17768699

[B39] Amato DJ A generalized Kaplan-Meier estimator for heterogeneous population Commun Stat Theory 1988 17 263 286 10.1080/03610928808829621

[B40] Ulm K Simple method to calculate the confidence interval of a standardized mortality ratio (SMR) Am J Epidemiol 1990 131 373 375 2296988 10.1093/oxfordjournals.aje.a115507

[B41] Ng HKT Tang ML Testing the equality of two Poisson means using the rate ratio Stat Med 2005 24 955 965 10.1002/sim.1949 15532090

[B42] Best JH Hoogwerf BJ Herman WH Pelletier EM Smith DB Wenten M Hussein MA Risk of cardiovascular disease events in patients with type 2 diabetes prescribed the glucagon-like peptide 1 (GLP-1) receptor agonist exenatide twice daily or other glucose-lowering therapies: a retrospective analysis of the LiveLink database Diabetes Care 2011 34 90 95 10.2337/dc10-1393 20929995 PMC3005487

[B43] UK Prospective Diabetes Study Group Tight blood pressure control and risk of macrovascular and microvascular complications in type 2 diabetes: UKPDS 38 BMJ 1998 317 703 713 9732337 PMC28659

[B44] UK Prospective Diabetes Study Group Efficacy of atenolol and captopril in reducing risk of macrovascular and microvascular complications in type 2 diabetes: UKPDS 39 BMJ 1998 317 713 720 9732338 PMC28660

[B45] Gaede P Lund-Andersen H Parving HH Pedersen O Effect of a multifactorial intervention on mortality in type 2 diabetes N Engl J Med 2008 358 580 591 10.1056/NEJMoa0706245 18256393

[B46] Schwartz TB Meinert CL The UGDP controversy: thirty-four years of contentious ambiguity laid to rest Perspect Biol Med 2004 47 564 574 10.1353/pbm.2004.0071 15467178

[B47] UK Prospective Diabetes Study Group Effect of intensive blood-glucose control with metformin on complications in overweight patients with type 2 diabetes (UKPDS 34) Lancet 1998 352 854 865 10.1016/S0140-6736(98)07037-8 9742977

[B48] Nathan DM Cleary PA Backlund JY Genuth SM Lachin JM Orchard TJ Raskin P Zinman B Intensive diabetes treatment and cardiovascular disease in patients with type 1 diabetes N Engl J Med 2005 353 2643 2653 10.1056/NEJMoa052187 16371630 PMC2637991

[B49] Holman RR Paul SK Bethel AM Matthews DR Neil HAW 10-Year follow-up of intensive glucose control in type 2 diabetes N Engl J Med 2008 359 1577 1589 10.1056/NEJMoa0806470 18784090

[B50] Diabetes Control and Complications Trial Research Group The effect of intensive treatment of diabetes on the development and progression of long-term complications in insulin-dependent diabetes mellitus N Engl J Med 1993 329 977 986 10.1056/NEJM199309303291401 8366922

[B51] Duckworth W Abraira C Moritz T Reda D Emanuele N Reaven PD Zieve FJ Marks J Davis SN Hayward R Warren SR Goldman S McCarren M Vitek ME Henderson WG Huang GD Investigators ftV Glucose control and vascular complications in veterans with type 2 diabetes New Engl J Med 2009 360 129 139 10.1056/NEJMoa0808431 19092145

[B52] Kelly TN Bazzano LA Fonseca VA Thethi TK Reynolds K He J Systematic review: glucose control and cardiovascular disease in type 2 diabetes Ann Intern Med 2009 151 394 403 19620144 10.7326/0003-4819-151-6-200909150-00137 PMC3058557

[B53] Bonds DE Miller ME Bergenstal RM Buse JB Byington RP Cutler JA Dudl RJ Ismail-Beigi F Kimel AR Hoogwerf B Horowitz KR Savage PJ Seaquist ER Simmons DL Sivitz WI Speril-Hillen JM Sweeney ME The association between symptomatic, severe hypoglycaemia and mortality in type 2 diabetes: retrospective epidemiological analysis of the ACCORD study BMJ 2010 340 b4909 10.1136/bmj.b4909 20061358 PMC2803744

[B54] Riddle MC Ambrosius WT Brillon DJ Buse JB Byington RP Cohen RM Goff DCJ Malozowski S Margolis KL Probstfield JL Schnall A Seaquist ER Epidemiologic relationships between A1C and all-cause mortality during a median 3.4-year follow-up of glycemic treatment in the ACCORD trial Diabetes Care 2010 33 983 990 10.2337/dc09-1278 20427682 PMC2858202

[B55] Stratton IM Adler AI Neil HA Matthews DR Manley SE Cull CA Hadden D Turner RC Holman RR Association of glycaemia with macrovascular and microvascular complications of type 2 diabetes (UKPDS 35): prospective observational study BMJ 2000 321 405 412 10.1136/bmj.321.7258.405 10938048 PMC27454

[B56] Selvin E Marinopoulos S Berkenblit G Rami T Brancati FL Powe NR Golden SH Meta-analysis: glycosylated hemoglobin and cardiovascular disease in diabetes mellitus Ann Intern Med 2004 141 421 431 15381515 10.7326/0003-4819-141-6-200409210-00007

[B57] Eeg-Olofsson K Cederholm J Nilsson PM Zethelius B Nunez L Gudbjörnsdóttir S Eliasson B Risk of cardiovascular disease and mortality in overweight and obese patients with type 2 diabetes: an observational study in 13,087 patients Diabetologia 2009 52 65 73 10.1007/s00125-008-1190-x 18985314

[B58] Maggio CA Pi-Sunyer FX The prevention and treatment of obesity. Application to type 2 diabetes Diabetes Care 1997 20 1744 1766 9353619 10.2337/diacare.20.11.1744

[B59] Nathan DM Buse JB Davidson MB Ferrannini E Holman RR Sherwin R Zinman B Management of hyperglycemia in type 2 diabetes: a consensus algorithm for the initiation and adjustment of therapy: update regarding thiazolidinediones: a consensus statement from the American Diabetes Association and the European Association for the Study of Diabetes Diabetes Care 2008 31 173 175 10.2337/dc08-9016 18165348

[B60] Mitri J Hamdy O Diabetes medications and body weight Expert Opin Drug Saf 2009 8 573 584 10.1517/14740330903081725 19538102

[B61] Rodbard HW Jellinger PS Davidson JA Einhorn D Garber AJ Grunberger G Handelsman Y Horton ES Lebovitz H Levy P Moghissi ES Schwartz SS Statement by an American Association of Clinical Endocrinologists/American College of Endocrinology consensus panel on type 2 diabetes mellitus: an algorithm for glycemic control Endocr Pract 2009 15 540 559 19858063 10.4158/EP.15.6.540

[B62] Gallwitz B Benefit-risk assessment of exenatide in the therapy of type 2 diabetes mellitus Drug Safety 2010 33 87 100 10.2165/11319130-000000000-00000 20082536

[B63] Buysschaert M Preumont V Oriot PR Paris I Ponchon M Scarnière D Selvais P Exenatide ftUSGf One-year metabolic outcomes in patients with type 2 diabetes treated with exenatide in routine practice. [French] Diabetes Metab 2010 36 381 388 10.1016/j.diabet.2010.03.009 20598606

[B64] Landstedt-Hallin L Englund A Adamson U Lins PE Increased QT dispersion during hypoglycaemia in patients with type 2 diabetes mellitus J Intern Med 1999 246 299 307 10.1046/j.1365-2796.1999.00528.x 10475998

[B65] Marques JL George E Peacey SR Harris ND Macdonald IA Cochrane T Heller SR Altered ventricular repolarization during hypoglycaemia in patients with diabetes Diabet Med 1997 14 648 654 10.1002/(SICI)1096-9136(199708)14:8<648::AID-DIA418>3.0.CO;2-1 9272590

[B66] Okerson T Yan P Stonehouse A Brodows R Effects of exenatide on systolic blood pressure in subjects with type 2 diabetes Am J Hypertens 2010 23 334 339 10.1038/ajh.2009.245 20019672

[B67] Buse JB Rosenstock J Sesti G Schmidt WE Montanya E Brett JH Zychma M Blonde L Liraglutide once a day versus exenatide twice a day for type 2 diabetes: a 26-week randomised, parallel-group, multinational, open-label trial (LEAD-6) Lancet 2009 374 39 47 10.1016/S0140-6736(09)60659-0 19515413

[B68] Bunck MC Cornér A Eliasson B Heine RJ Shaginian RM Wu Y Yan P Smith U Yki-Järvinen H Diamant M Taskinen MR One-year treatment with exenatide vs. Insulin Glargine: Effects on postprandial glycemia, lipid profiles, and oxidative stress Atherosclerosis 2010 212 223 229 10.1016/j.atherosclerosis.2010.04.024 20494360

[B69] Bunck MC Diamant M Eliasson B Cornér A Shaginian RM Heine RJ Taskinen MR Yki-Järvinen H Smith U Exenatide affects circulating cardiovascular risk biomarkers independently of changes in body composition Diabetes Care 2010 33 1734 1737 10.2337/dc09-2361 20424219 PMC2909051

[B70] Anselmino M Öhrvik J Rydén L Resting heart rate in patients with stable coronary artery disease and diabetes: a report from the euro heart survey on diabetes and the heart Eur Heart J 2010 31 3040 3045 10.1093/eurheartj/ehq368 20935002

[B71] Stettler C Bearth A Allemann S Zwahlen M Zanchin L Deplazes M Christ ER Teuscher A Diem P QT_c _interval and resting heart rate as long-term predictors of mortality in type 1 and type 2 diabetes mellitus: a 23-year follow-up Diabetologia 2007 50 186 194 10.1007/s00125-006-0483-1 17096116

[B72] Linnebjerg H Seger M Kothare P Hunt T Hons B Mitchell MI The effect of exenatide on QTc interval in healthy subjects [abstract] Diabetes 2009 58 A161

[B73] Nikolaidis LA Elahi D Hentosz T Doverspike A Huerbin R Zourelias L Stolarski C Shen YT Shannon RP Recombinant glucagon-like peptide-1 increases myocardial glucose uptake and improves left ventricular performance in conscious dogs with pacing-induced dilated cardiomyopathy Circulation 2004 110 955 961 10.1161/01.CIR.0000139339.85840.DD 15313949

[B74] Zhao T Parikh P Bhashyam S Bolukoglu H Poornima I Shen Y Shannon R Direct effects of glucagon-like peptide-1 on myocardial contractility and glucose uptake in normal and postischemic isolated rat hearts J Pharmacol Exp Ther 2006 317 1106 1113 10.1124/jpet.106.100982 16489128

[B75] Bose AK Mocanu MM Carr RD Brand CL Yellon DM Glucagon-like peptide 1 can directly protect the heart against ischemia/reperfusion injury Diabetes 2005 54 146 151 10.2337/diabetes.54.1.146 15616022

[B76] Liu Q Adams L Broyde A Fernandez R Baron AD Parkes DG Liu Q Adams L Broyde A Fernandez R Baron AD Parkes DG The exenatide analogue AC3174 attenuates hypertension, insulin resistance, and renal dysfunction in Dahl salt-sensitive rats Cardiovasc Diabetol 2010 9 32 10.1186/1475-2840-9-32 20678234 PMC2922097

[B77] Liu Q Anderson C Broyde A Polizzi C Fernandez R Baron A Parkes DG Glucagon-like peptide-1 and the exenatide analogue AC3174 improve cardiac function, cardiac remodeling, and survival in rats with chronic heart failure Cardiovasc Diabetol 2010 9 76 10.1186/1475-2840-9-76 21080957 PMC2996354

[B78] Nikolaidis LA Mankad S Sokos GG Miske G Shah A Elahi D Shannon RP Effects of glucagon-like peptide-1 in patients with acute myocardial infarction and left ventricular dysfunction after successful reperfusion Circulation 2004 109 962 965 10.1161/01.CIR.0000120505.91348.58 14981009

[B79] Sokos GG Nikolaidis LA Mankad S Elahi D Shannon RP Glucagon-like peptide-1 infusion improves left ventricular ejection fraction and functional status in patients with chronic heart failure J Card Fail 2006 12 694 699 10.1016/j.cardfail.2006.08.211 17174230

[B80] Eldor R Kidron M Greenberg-Shushlav Y Arbit E Novel glucagon-like peptide-1 analog delivered orally reduces postprandial glucose excursions in porcine and canine models J Diabetes Sci Technol 2010 4 1516 1523 21129350 10.1177/193229681000400629 PMC3005065

